# Innate and adaptive immunity in the development of depression: An update on current knowledge and technological advances

**DOI:** 10.1016/j.pnpbp.2015.11.012

**Published:** 2016-04-03

**Authors:** Rita Haapakoski, Klaus P. Ebmeier, Harri Alenius, Mika Kivimäki

**Affiliations:** a Department of Epidemiology and Public Health, University College London, United Kingdom; b Department of Psychiatry, University of Oxford, United Kingdom; c Finnish Institute of Occupational Health, Systems Toxicology Unit, Helsinki, Finland; d Department of Public Health, Faculty of Medicine, University of Helsinki, Finland

**Keywords:** Depression, Inflammation, Cytokine, Innate immunity, Adaptive immunity, Omics technologies

## Abstract

The inflammation theory of depression, proposed over 20 years ago, was influenced by early studies on T cell responses and since then has been a stimulus for numerous research projects aimed at understanding the relationship between immune function and depression. Observational studies have shown that indicators of immunity, especially C reactive protein and proinflammatory cytokines, such as interleukin 6, are associated with an increased risk of depressive disorders, although the evidence from randomized trials remains limited and only few studies have assessed the interplay between innate and adaptive immunity in depression. In this paper, we review current knowledge on the interactions between central and peripheral innate and adaptive immune molecules and the potential role of immune-related activation of microglia, inflammasomes and indoleamine-2,3-dioxygenase in the development of depressive symptoms. We highlight how combining basic immune methods with more advanced ‘omics’ technologies would help us to make progress in unravelling the complex associations between altered immune function and depressive disorders, in the identification of depression-specific biomarkers and in developing immunotherapeutic treatment strategies that take individual variability into account.

## Highlights

• Depressive patients have often (but not always) alterations in innate and adaptive immunity.
• Depression-related immunological changes interact with neurohormonal circuits.
• Increased production of inflammasomes and neurotoxic catabolites may be part of this process.
• Future research on immunity and depression will benefit from novel omics techniques.

## Graphical abstract

**Figure:**
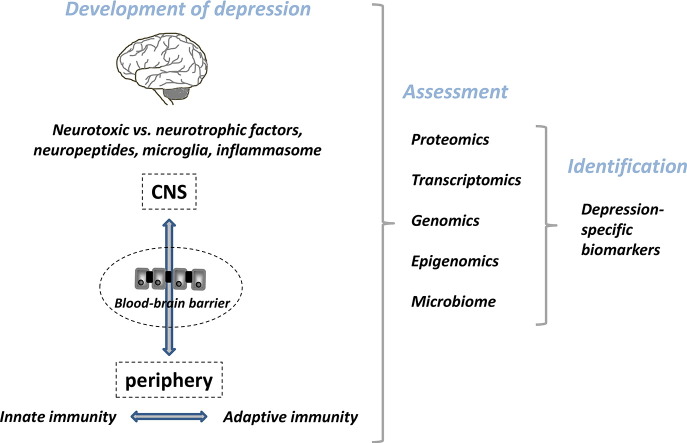

## 1 Introduction

The immune system consists of biological structures and processes that help the organism adapt to physiological or psychological stressors. The outcome, inflammation, is a part of this system. It is a biological host defence mechanism characterized by increased blood flow and recruitment of innate immune cells to the site of injury. The link between increased inflammation and depression was detected in the early 1990s (Maes et al., 1990, Maes et al., 1991), leading to the formulation of the macrophage hypothesis of depression (also known as the cytokine hypothesis of depression (Maes, 1995, Smith, 1991). This model proposes that external and internal stressors trigger depressive behaviour by elevating the production of proinflammatory cytokines interleukin-1 (IL-1) and IL-6, as well as activating cell-mediated immunity. More recently, an abundance of observational, experimental and clinical evidence has emerged to suggest that the activation of innate immune mechanisms, especially proinflammatory cytokines IL-1, IL-6 and tumor necrosis factor alpha (TNF-α), as well as C-reactive protein (CRP), may contribute to the initiation and progression of psychiatric diseases, such as depression (Capuron et al., 2003, Dowlati et al., 2010, Gimeno et al., 2009, Howren et al., 2009, Kivimaki et al., 2014, Liu et al., 2012, Raison et al., 2010, Valkanova et al., 2013). Several recent publications have focused on these associations (Capuron and Miller, 2011, Dantzer et al., 2008, Haroon et al., 2012, Jones and Thomsen, 2013, McCusker and Kelley, 2013, Mills et al., 2013, Mitchell and Goldstein, 2014, Quan and Banks, 2007, Raedler, 2011, Rivest, 2009) and while the majority of this evidence involves pro-inflammatory cytokines and CRP, changes in the function and numbers of innate immune cells, namely natural killer (NK) cells, have also been examined.

In addition to increased innate immune responses, activation of cell-mediated adaptive immunity has been described in depressed patients. This includes increased CD4 +/CD8 + T cell ratios, i.e. higher percentage of CD4 + T cells and a lower percentage of CD8 + T suppressor cells (Darko et al., 1988, Maes et al., 1992b, Tondo et al., 1988). Furthermore, elevated numbers and a higher proportion of activated T cells bearing activation markers CD2 + CD25 +, CD3 + CD25 +, HLA-DR + (Maes et al., 1992a), higher blood levels of IL-2R (Liu et al., 2012) and increased number of B cell subsets (Maes et al., 1992c, Robertson et al., 2005) have been detected in patients with major depressive disorder compared with controls. On the other hand, reduced proliferative response of T cells to mitogen in subjects with depression has been shown in a meta-analysis (Zorrilla et al., 2001), suggesting that depression may be associated with concurrent activation and suppression of immune responses.

In this review, we present a model on co-operative mechanisms between innate and adaptive immunity in the periphery and central nervous system (CNS), and the potential role of such molecules in the pathology of depressive mood. We discuss the interrelations between cytokines, T cells, NK cells, inflammasomes, microglia, indoleamine-2,3-dioxygenase (IDO) and neurotoxic vs. neurotrophic factors, all of which have been suggested to contribute to the initiation and progression of depressive symptoms. Finally, we discuss how novel methodologies, such as the ‘omics’ technologies, might represent the next step in uncovering the relations between altered immune function and depressive disorders.

## 2 Evidence on immune–brain relations

In animal experiments, peripherally administered proinflammatory cytokines IL-1β and TNF-α as well as lipopolysaccharide (LPS) and synthetic compound mimicking viral infection (Poly (I:C)) have induced ‘sickness behaviour’ characterized by lethargy, depression, anxiety, loss of appetite, and sleepiness (Dantzer, 2001, Gibney et al., 2013). This response is thought to be caused by pro-inflammatory cytokines temporarily expressed in the brain during infection (Dantzer et al., 2008). In humans, injections with LPS (Reichenberg et al., 2001) or administration of typhoid vaccine (Harrison et al., 2009) have been shown to be related to an increased production of proinflammatory cytokines and subsequent decline in mood, while pre-treatment with an antidepressant drug, citalopram, a selective serotonin reuptake inhibitor, has reduced LPS-induced depressive symptoms in healthy subjects (Hannestad et al., 2011).

Information on brain-immune relations in the CNS has also been acquired using positron emission tomography (PET) to measure the density of translocator protein (TSPO), a microglia-derived protein showing increased expression during neuroinflammation. In one PET study, TSPOV_T_ (volume distribution) was elevated in patients with more severe forms of depression (Setiawan et al., 2015). Another study failed to find differences in the levels of [^11^C]PBR28VT, a ligand binding to TSPO, between individuals with mild-to-moderate depression and control subjects (Hannestad et al., 2013). These results suggest that neuroinflammatory activation via TSPO may only be observed in more severe forms of depression, but more research is needed to confirm these relations. Furthermore, cross-sectional design of these studies cannot inform about whether inflammation is a cause of a consequence of depression.

Additional evidence for immune–brain associations has emerged from clinical studies. For example, approximately 20–50% of cancer and hepatitis C patients treated with injections of interferon-α (IFN-α) have been estimated to develop clinically significant depression (Raison et al., 2005). IFN-α-induced depression has been associated with elevated serum levels of sIL-2r, TNF-α, and IL-6 (Wichers et al., 2007). Interestingly, there is also evidence to suggest that this IFN-α induced depression is responsive to conventional antidepressant treatments, an observation consistent with the hypothesized shared pathways between inflammation and idiopathic major depression (Capuron et al., 2002, Musselman et al., 2001).

Epidemiological studies provide further support for the association between altered inflammatory profile and depression. A recent cumulative meta-analysis on 31 cross-sectional studies on IL-6 and 20 studies on CRP showed a robust association between increased levels of these two inflammatory markers and major depression, although the relations between TNF-α and major depression were not confirmed in a total of in 31 studies due to extensive heterogeneity in study-specific effect estimates and inconsistencies between subgroups (Haapakoski et al., 2015). No consistent evidence support a link between IL-1β and depression (14 studies), which may in part be related to the very low concentrations of this cytokine in peripheral blood and a lack reliable detection methods. At least two meta-analyses support the existence of reduced proliferative activity of lymphocytes and lowered NK cell activity in depression (Herbert and Cohen, 1993, Zorrilla et al., 2001). Furthermore, increased number of leukocytes has been associated with depression (Zorrilla et al., 2001) whereas evidence regarding the number of different subsets of T cells, B cells, NK cells and NKT cells in depression is inconclusive. These findings suggest specificity in the associations of various inflammatory markers and depression but several methodological issues may also contribute to the observed inconsistencies, including the heterogeneity of depressive disorder and imprecise measurement of some subsets of immune cells.

Longitudinal analyses of observational data provide inconsistent information on the temporal order between CRP, proinflammatory cytokines and depression. In the Whitehall II study, for example, high levels of CRP and IL-6 at baseline have been associated with an increased risk of future cognitive symptoms of depression whereas baseline symptoms of depression did not predict the level of CRP or IL-6 at follow-up (Gimeno et al., 2009). This finding is in agreement with a study showing that elevated levels of IL-6 in childhood are associated with an increased risk of depression in young adulthood (Khandaker et al., 2014). Chronically elevated inflammation in the development of psychiatric symptoms is supported by data from Whitehall II study showing repeated measurements of IL-6 dose-dependently increasing the risk of future common mental disorder (Kivimaki et al., 2014). On the other hand, at least one study has found that somatic-vegetative symptoms of depression predicted 6-year change in IL-6 levels whereas baseline levels of neither IL-6 nor CRP were predictors of change in depressive symptoms in older individuals (Stewart et al., 2009). In another study, depressive symptoms predicted CRP levels in adolescents and young adults (Copeland et al., 2012). These results indicate the possibility of bidirectional association between inflammation and depression and that the strength and direction of the association may be dependent on specific depressive symptom clusters, type of immune markers measured and/or population characteristics such as age, patient type (communal vs. clinical sample) and comorbid disorders. More prospective investigations including repeat measurements of both immune function and depression in different patient groups and with various immune markers are needed to ascertain the extent to which inflammation is a cause and a consequence of depression.

## 3 A model of immune–brain interactions in mood regulation

**Fig. 1:**
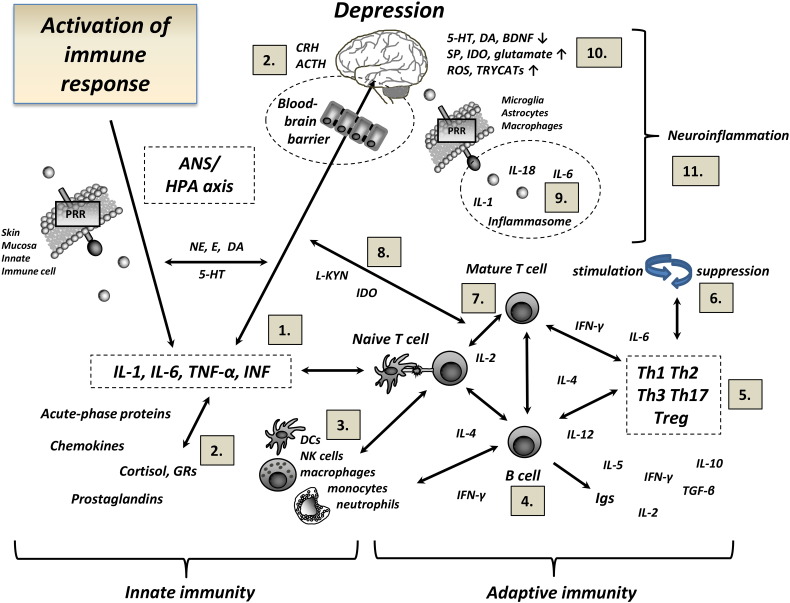
Suggested links of innate and adaptive immunity and neuroendocrine circuits with the development of depression. Activation of immune response and the production of proinflammatory cytokines, chemokines and acute phase proteins may be induced by external exposure to bacterial or viral components or via psychological or physiological stress reaction, exercise, inflammation, trauma or tissue injury. Cross talk between innate and adaptive immune cells, inflammatory and endocrine signalling molecules and neuromodulatory processes in the periphery and the central nervous system may have a profound effect on behavioural alterations and the development of depression. Numbers in the figure designate key references related to specific immune–brain associations (references list and explanation for abbreviations are provided in the Supplementary material).

Fig. 1 illustrates a simplified model where interactions between innate and adaptive immune mechanisms and neuronal and hormonal connections, both in the periphery and the brain, provide excitatory and suppressive signals leading to the activation of multiple brain regions involved in mood regulation. Immune response can be initiated by external exposure to bacterial or viral components, such as lipopolysaccharide (LPS) or synthetic dsRNA Poly (I:C) or via psychological or physiological stress reaction, vigorous exercise, inflammation, trauma or tissue injury. In the periphery, different pattern recognition receptors (PRRs, e.g. Toll like receptors [TLRs] and C-lectin like receptors [CLRs]) are expressed in the outer membranes of the skin or mucosa and in the cells of innate immunity such as NK cells and dendritic cells (DCs) (referred also as antigen presenting cells; APCs) providing the first contact of the micro-organism to the body's cells (Janeway and Medzhitov, 2002). Binding of ligand to its specific receptor leads to the activation of signalling transduction cascades and to the production of pro-inflammatory cytokines, IL-1β, TNF-α and IFN, chemokines and acute phase proteins. Furthermore, stress response activates mechanisms within the sympathetic nervous system (SNS) and the hypothalamo-pituitary-adrenal (HPA) axis associated with increased production of catecholamines, such as norepinephrine (NE) and epinephrine (E), and the glucocorticoid cortisol in the blood. Neuropeptide Y (NPY) is also released from the sympathetic nerve terminals in response to exposure to stressors.

Proinflammatory cytokines IL-1, IL-6 and TNF released in response to a stressor activate the HPA axis and induce the release of endogenous glucocorticoids which, in turn, suppress HPA activity and innate immune responses. Constant communication and feedback mechanism between corticotrophin-releasing hormone (CRH), adrenocorticotropic hormone (ACTH), cortisol and catecholamines E and NE contribute to the regulation of hormonal and immunological responses in the periphery and the CNS and to physiological response to stress (Black, 2002, Ulrich-Lai and Herman, 2009). The increased production of proinflammatory cytokines and immune intermediates is thought to lead to impairments in glucocorticoid receptor signalling and glucocorticoid resistance, conditions frequently observed in depressed patients (Pace and Miller, 2009). Furthermore, E and NE have been shown to affect lymphocyte traffic, migration, and proliferation as well as cytokine production (Elenkov et al., 2000), supporting neuroendocrine communication between the immune system and the brain.

### 3.1 The bridge between innate and adaptive immunity in depression

Fig. 1 also shows that interactions between the innate and adaptive immune systems in the periphery and the CNS may result in stimulation or suppression of T cells and NK cells, both phenomena being demonstrated in depressed patients (Liu et al., 2012, Maes, 1995, Maes et al., 1992a, Pavon et al., 2006, Robertson et al., 2005, Zorrilla et al., 2001). In addition, there is evidence on altered Th1/Th2 cytokine and regulatory T cell (Treg) balance in patients with major depression (Li et al., 2010, Pavon et al., 2006). In the periphery, DCs play a critical role in mediating the communication between the innate and adaptive immunity by acting as professional APCs, activating CD4 +/CD8 + T cells and regulating T lymphocyte function (Merad et al., 2013, Steinman, 2007). In the CNS, pathogen-associated molecular patterns (PAMPs) or danger-associated molecular patterns (DAMPs) regulate the production of pro-inflammatory cytokines IL-1β, IL-6 and TNF-α and activation of T lymphocytes (Shastri et al., 2013). The availability of PRRs/PAMPs/DAMPs and co-stimulatory molecules in CNS-resident cells and the overall nature of the cytokine milieu provided by different APCs have been suggested to serve as a bridge between innate and adaptive immune cascades and as determinants of T cell differentiation (Steinman, 2007). There is also emerging evidence that NK cells contribute to long-lasting “memory-like” functional alterations indicating that NK cells may play an important role as a bridge between innate and adaptive immunity (Rolle et al., 2013).

Immune-inducing molecules can activate brain signalling via a variety of neural, humoral and cellular mechanisms. For example, cytokines may enter into the brain parenchyma passively through the leaky regions in the blood brain barrier (BBB) such as the choroid plexus (CP), by using cytokine-specific transport molecules or by transmission of cytokine signals via afferent vagus nerve fibers (reviewed in (Capuron and Miller, 2011, Dantzer, 2009, Dantzer et al., 2008). Mature T lymphocytes are able to enter the CNS via several routes during conditions of stress and in disease states (Ransohoff et al., 2003). Immune cells can be found at least in three anatomical sites in the brain in normal physiological and in disease states, that is, in CSF, meninges and brain parenchyma (Wilson et al., 2010).

In murine models, the traffic of T cells to the CP following exposure to a psychological stress, and immunization with CNS-specific peptide activating self-reactive T cells have been reported to modify depressive behaviour; this effect seems to be accompanied by a restoration of the levels of brain-derived neurotrophic factor (BDNF) to the pre-stress level (Lewitus et al., 2008, Lewitus et al., 2009) and the development of new neurons (neurogenesis) in the hippocampus (Lewitus et al., 2009). The importance of lymphocytes in protecting the host from psychological stress reaction is supported by recent study showing less anxiety, reduced pro-inflammatory cytokine levels and increased neuroprotective profile in mice receiving lymphocytes from defeated donors compared with those receiving cells from unstressed donors (Brachman et al., 2015). Furthermore, decreased circulating BDNF levels in patients with depression (Molendijk et al., 2014) and positive associations between elevated BDNF and IL-6 levels and between BDNF and leukocyte counts in patients with major depression (Patas et al., 2014) suggest that interactions between T and B cells, cytokines and neurotrophic factors, such as BDNF, may be associated with the neuropathology of depression.

Deficits in NK cell function have been hypothesized to be associated with the pathology of depression (Herbert and Cohen, 1993, Zorrilla et al., 2001). Reduced NK cell activity in combination with an increased serum IL-6 level is indicative of the co-existence of suppression and activation of innate immune responses in depression (Blume et al., 2011, Pike and Irwin, 2006). Deficits in NK-cell activity have also been associated with symptoms frequently associated with mood disorders, such as sleep disturbances (Irwin et al., 1994, Irwin et al., 1996). In line with this concept, sleep restriction has increased lymphocyte activation, elevated the numbers of B cells as well as increased the production of proinflammatory cytokines IL-1β, IL-6, IL-17 and CRP and at the same time reduced the number of NK cells (van Leeuwen et al., 2009). Furthermore, acute psychophysiological stress has been associated with blunted levels of cortisol, increased levels of IL-6 and greater percentage of Treg cells (Ronaldson et al., 2015).

Exposure to acute stress reaction such as sleep disturbances, a phenomenon commonly found in melancholic depression, leads to blunted activation and number of APCs (DCs and NK cells) and increased proinflammatory cytokines and CRP. Consequently, the number of T and B cells may temporarily increase to prepare the body to fight against “intruder”. When the stress reaction sustains, as in chronic depression, these mechanisms may become maladaptive and detrimental; i.e. reduced availability of messenger cells for T cell activation may result in lowered stimulatory activity of leukocytes and down regulation of body's defence mechanisms. Furthermore, lowered numbers of Treg cells in depressed patients (Li et al., 2010) suggest an imbalance between positive and negative regulatory mechanisms after chronic exposure to stressor. Especially in vulnerable individuals, these reactions may be associated with increased susceptibility to physical and physiological harm and worsening of depressive symptoms.

### 3.2 Role of microglia and inflammasomes

In the CNS, microglia cells residing in the brain parenchyma represent the chief innate immune cells capable of mediating innate and adaptive immune responses via PRR-mediated recognition (Rivest, 2009) (Fig. 1). Microglial cells can engulf and clear damaged cell material and phagocytose neurons undergoing apoptosis (Nimmerjahn et al., 2005). Microglia also has an important role in regulating synaptic pruning during brain development (Paolicelli et al., 2011). PRRs expressed in microglia cells, astrocytes and CNS-derived macrophages act as sensors for foreign antigens by stimulating the migration of immune cells, e.g. macrophages, neutrophils, T cells and B cells to the site of injury and by activating astrocytes and adjacent glial cells (Beynon and Walker, 2012, Walsh et al., 2014).

The intensity and nature of the effects mediated by inflammation-activated microglia may differ depending on the identity and combination of activated TLRs (Rosenberger et al., 2014). For example, endogenous DAMP produced in response to non-physiological cell death, damage or stress reaction are able to activate innate immune cells via TLR mediated signalling and induce the formation of the inflammasome consisting of multiprotein complexes formed by NLR family members NLRP1, NLRP3, and NLRC4 (Bianchi, 2007, Leemans et al., 2011). These danger signals are thought to function synergistically with PAMPs to alert the immune system to rapidly mobilize the release of their intracellular contents into the extracellular space and to provide the initial defence against pathogens and damage control of tissue injury (Kono and Rock, 2008). Initiation of an inflammatory response and the formation of the inflammasome enable the activation of proinflammatory caspases, mainly caspase-1 and subsequently, increased release of pro-inflammatory cytokines (Walsh et al., 2014). A recent review has proposed an integrated role of glucocorticoids and NLRP3-mediated inflammation in response to stress (Frank et al., 2013). According to this model, the effects of glucocorticoids on TLRs and inflammasomes prime and facilitate the central and peripheral inflammatory immune responses via immune effectors (e.g. microglia) during stress (fight/flight) response in situations of exogenous injury.

Preclinical models suggest a role for immune-sensing microglia cells in the development of depressive-like behaviour (Fenn et al., 2014, Kreisel et al., 2014). This is consistent with the possibility that inflammasome-mediated caspase-1 activation and induction in the production of cytokines IL-1β and IL-18 in microglia cells are an important mechanism mediating the pathological processes in neuropsychiatric disorders associated with elevated inflammation. In support of this concept, increased gene expression of NLRP3 and caspase-1 in the mononuclear blood cells and elevated serum levels of IL-1β and IL-18 have been found in patients with major depression (Alcocer-Gomez et al., 2014). Furthermore, NLRP3 has been associated with LPS-induced depression in a murine model (Zhang et al., 2014), suggesting a role of inflammasomes and Nod-like receptor signalling pathways in the immunity–depression relationship.

### 3.3 Role of IDO, glutamate and neurotoxic catabolites

Recently, the role of enzyme IDO in the immunity-depression linkage has received increasing attention (Chen, 2011). Activation of IDO is induced by the production of proinflammatory cytokines as a response to physiological or psychological stress. IDO is the enzyme that catalyzes the rate-limiting step in the degradation of tryptophan (TRP), an essential amino acid in the conversion of serotonin into kynurenine (KYN). KYN is further metabolized into tryptophan catabolites (TRYCATs) such as neuroprotective kynurenine acid (KA) and neurotoxic quinolinic acid (QUIN). QUIN, primarily produced by brain microglial cells, is an agonist of the glutamatergic N-methyl-d-aspartate (NMDA) receptor; therefore, activation of IDO leads to a reduced synthesis of serotonin as well as to the overactivation of glutamate receptors in the brain (these processes have been described in detail by Dantzer et al. (2008) and in (Muller and Schwarz, 2007). Consequently, sustained release of proinflammatory cytokines by microglial cells, called neuroinflammation, may promote neuronal damage and neuronal death, similar to decreased neurogenesis and reduced serum levels of BDNF and increased glutamate release or inhibition of glutamate uptake (Pav et al., 2008, Tilleux and Hermans, 2007). Several tryptophan metabolites and glutamate are neurotoxic and this may result in neuronal damage and cellular loss especially in the hippocampus, potentially increasing the risk of neuropsychiatric disorders (Maes et al., 2011, Myint et al., 2012).

The role of neurotoxic metabolites in immune-modulated neurotransmission is supported by studies showing upregulated levels of peripheral and central KYN and increased QUIN in the CNS, correlating with IFN-α induced depression (Raison et al., 2010). Further evidence of the role of IDO and KYN in depression comes from preclinical studies showing an induction of depression-like behaviour after administration of l-kynurenine (l-KYN) (O'Connor et al., 2009). Furthermore, in some studies depressed patients have had an increased density of QUIN-positive cells (Steiner et al., 2011) increased glutamate (Hashimoto et al., 2007) and deficits in glutamate transporter molecules in the brain cortex (Choudary et al., 2005). Despite reduced levels of peripheral TRP, the central levels of TRP do not appear to be affected by IFN-a treatment (Raison et al., 2010), raising the possibility that neurotoxic end-products rather than serotonin play a role in the pathology of depression.

Microarray analyses of post-mortem frontal cortex provide some evidence on increased inflammatory, apoptotic, and oxidative stress in patients with major depressive disorder (Shelton et al., 2011). For example, the serum kyurenic acid (KA)/quinolinic acid (QUIN) ratio, a putative neuroprotective index, tended to be lower in patients with major depression compared to those free of depression and this ratio has been found to be associated with higher hippocampal and amygdala volumes (Savitz et al., 2015a, Savitz et al., 2015b). However, the evidence suggesting morphological alterations in depression is relatively modest and the overall change in neuronal loss in depression has been suggested to be rare (Lucassen et al., 2001) requiring exposure to prolonged severe stress before changes become detectable (Lucassen et al., 2006). Meta-analytic reviews have found discrepancies regarding the volumetric differences in various brain areas in depressed versus control subjects with, for example, lower hippocampal volume in depression being detectable only when measured as a discrete structure in patients with longstanding illness (Campbell et al., 2004) or in patients with more than one previous disease episode (McKinnon et al., 2009). In addition, a meta-analytic review concluded that although late-life depression appears to be associated with minor volume reductions in hippocampus in the summary evidence, this association remains uncertain as there was heterogeneity between studies with many studies reporting negative findings (Sexton et al., 2013). These results indicate that while there is some evidence supporting the involvement of neurotoxic catabolites KYN and QUIN in the pathology of depression, more research is needed to conclude whether activation of IDO and involvement of neurodegenerative substances are associated with hippocampal atrophy, reduced neurogenesis and neuronal cell death in depressive individuals.

### 3.4 Associations between inflammatory and neuroendocrine mechanisms in depression

Extensive evidence shows an association of inflammation with HPA axis malfunction and bi-directional associations between immune and neuroendocrine systems (Maes et al., 1993, Maes et al., 2009, Raison and Miller, 2003, Tafet and Bernardini, 2003, Zunszain et al., 2011). Alterations in both cellular (Th1) and humoral (Th2) arms of adaptive immunity have been linked to the HPA axis function; i.e. glucocorticoids suppress the production of Th1 type cytokine IL-12 but upregulate the production of IL-4 by Th2 cells (Blotta et al., 1997, Franchimont et al., 2000). Conversely, cytokines are capable of stimulating the HPA axis directly, as indicated by the enhanced release of ACTH and glucocorticoids in patients developing depressive symptoms after IFN-α–treatment (Capuron et al., 2003). Cytokines are thought to be involved in the impaired feedback mechanisms and glucocorticoid resistance via their signalling pathways, such as nuclear factor-κB (NF-κB,) signal transducers and activators of transcription, all of which have been found to inhibit GR function (Pace et al., 2007). However, considerable variation in the degree of HPA activation in depressed patients exists and some patients display no evidence of HPA overactivity (Marques-Deak et al., 2007, Strickland et al., 2002, Swaab et al., 2005, Watson et al., 2002). This may partly reflect the heterogeneity of depressive disorders, with the role of HPA axis dysfunction being more prominent in the more severe forms of the disease, such as in melancholic and psychotic depression (Lamers et al., 2013, Nelson and Davis, 1997, O'Keane et al., 2012). In addition, it has been suggested that interactions between inflammatory molecules, glucocorticoids and other HPA axis mechanisms, such as renin–angiotensin–aldosterone system (RAAS), nutritional gut hormone glucagon-like peptide 1 and growth hormones, may be associated with the development of depressive symptoms and contribute to the high prevalence of immune-related chronic disorders, such as diabetes, in groups of depressive patients (Mahajan et al., 2004, Murck et al., 2012, Numakawa et al., 2013).

### 3.5 Summary of evidence

Preclinical and clinical evidence shows both activation and suppression of immune responses in depressed patients. While an abundance of evidence supports increased levels of proinflammatory cytokines and acute phase proteins in depression, important questions still remain regarding the role of altered adaptive immunity and the co-operation between innate and adaptive immune mechanisms in depressive disorders. Among the many fundamental questions that remain unanswered are the “protective autoimmunity” mediated by different, yet unknown subsets of CNS autoreactive T cells (Miller, 2010, Rook et al., 2011). These T cells have shown neuroprotective and anti-depressive effects in preclinical models, indicating that T cells may possess some, yet unknown, role in maintaining homeostatic functions in depression. Furthermore, the specific role of different T cell subsets, e.g. regulatory T cells and CD4 +/CD8 + T cells in the pathology of depression are yet to be established.

It has been speculated that increased neuronal activity triggers innate and adaptive immune cells, vascular cells and neurons in the CNS leading to pathological neuroinflammation after persistent exposure to the stressors (Xanthos and Sandkuhler, 2014). Increasing evidence also postulates the activation of microglia and the inflammasome-mediated pathways in the pathology of depression (Setiawan et al., 2015). In particular, activation of NLRP inflammasomes and increased proinflammatory cytokines, including IL-1β, is suggested to be involved in the maladaptive changes in the CNS during depressive episode. Another hypothesis linked to immune-induced depression, besides the well-known serotonin deficiency, is an increased activation of IDO and accumulation of glutamate and tryptophan catabolites such as KYN. KYN is known to possess many immunomodulatory effects on innate and adaptive immune cells, such as a blockade of T and NK cell proliferation and induction of T cell apoptosis (Fallarino et al., 2003, Terness et al., 2002). Since peripheral l-KYN and 3-hydroxy-l-kynurenine (3-HK) can enter the CNS via the blood brain barrier (Vecsei et al., 2013), central and/or peripheral activation of IDO and the formation of neurotoxic catabolites in response to increased inflammatory activity may account for the adverse innate and adaptive immune effects and the perpetuation of other central immune-induced changes, such as serotonin deficiency associated with depression (Dantzer et al., 2011). Reduced production of factors linked to neuronal plasticity and cell growth, such as BDNF, may further increase the risk for depressive symptoms in susceptible individuals (Bocchio-Chiavetto et al., 2010).

During the acute phase of stress, proinflammatory cytokines, acute phase proteins, chemokines and other inflammatory mediators and the activation status of APCs and NK cells increase. In addition, the immune-induced processes such as formation of inflammasomes and increased production IDO are activated via CNS resident astrocytes, macrophages or microglia. Chronic elevations in proinflammatory cytokines, and imbalance in pro- and anti-inflammatory cytokines, changes in the number and activity of T and B cells, development of glucocorticoid resistance and the accumulation of cell-toxic and neurodegenerative elements may lead maladaptive changes when the body's natural coping mechanisms fail to maintain homeostasis and respond efficiently to external stressors. Depending on comorbid diseases, overall health status and other risk factors these pathological processes may induce worsening of physiological symptoms and accelerate the development of depression in certain individuals. In the future, the challenge lies in identifying subgroups of depressed patients with increased inflammatory-induced symptoms, who could benefit from immunomodulatory anti-depressive therapies.

## 4 Omics technologies in depression research

**Fig. 2:**
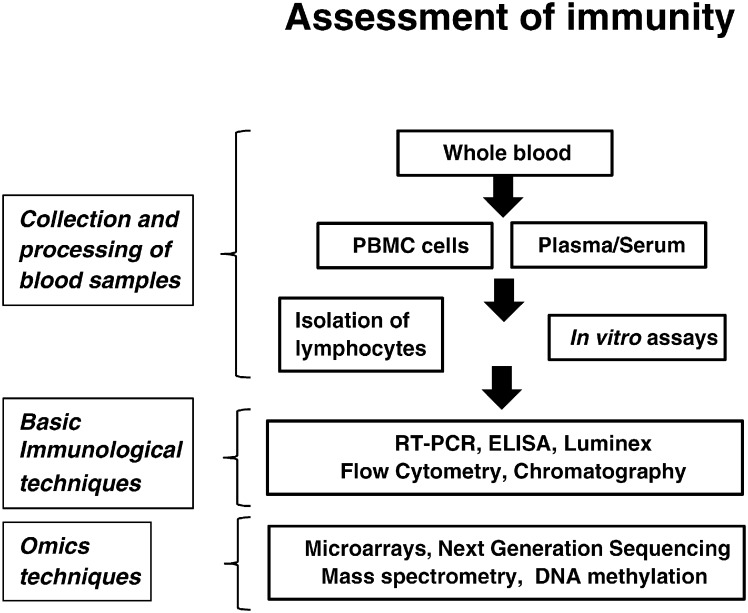
Examples of basic immunological methodologies and high-throughput “omics” techniques for the investigation of immune mechanisms in depressive patients using peripheral blood. Abbreviations: PBMC: peripheral blood mononuclear cell; RT-PCR: real-time polymerase chain reaction; ELISA: enzyme-linked immunosorbent assay.

Despite substantial research efforts, the exact role of immunological status in depression pathology has remained elusive. To advance this research, a number of immunological techniques, ranking from basic laboratory techniques to more sophisticated sample analysis involving omics techniques (proteomics, transcriptomics, metabolomics, genomics and epigenomics) may be used to study the integrated networks of genes, proteins and biochemical interactions in the periphery and in the CNS (Fig. 2). Combining immunological data with neuropsychiatric tests and clinical assessments may provide a better outlook on the hormonal and structural alterations associated with depression.

Omics technologies could, for example, aid in the identification of new, depression-specific biomarkers which may be applied for several purposes, including improvement of diagnosis, predicting the course of disease and designing more personalized treatment methods for depression (Domenici et al., 2010, Schwarz and Bahn, 2008, Uher et al., 2012). Biomarker studies may use two different approaches:

(a) A hypothesis-driven method with pre-selected biomarkers and

(b) a hypothesis-free, data-driven method.

Inflammatory molecules, in particular proinflammatory cytokines, provide an example of hypothesis-driven biomarkers with respect to various depression models, including the vascular depression concept as the inflammatory markers have been related to an increased risk for future cardiovascular diseases (Engstrom et al., 2004, Galea and Brough, 2013, Hingorani and Casas, 2012, Ridker et al., 2000) and depression (Gimeno et al., 2009, Kivimaki et al., 2014, Lanquillon et al., 2000). For example, elevated levels of inflammatory biomarkers CRP, IL-1β and TNF-α have been linked to poor antidepressant treatment response in depressed patients (Cattaneo et al., 2013, Uher et al., 2014), indicating that inflammatory molecules may have role as predictive biomarkers in depression treatment, for example in estimating the efficacy or suitability of a treatment before the trial.

Recent technological advances have triggered many new research projects utilizing data-driven profiling assays where no prior knowledge of genes involved in the process is required. Omics technologies such as mass spectrometry, microarray or sequencing methods may be especially beneficial in the search for biomarkers for the identification of disease-specific markers for depression (Ditzen et al., 2012, Filiou et al., 2012, Sunde, 2010). This approach allows researchers to investigate thousands of potential gene transcripts of unknown origin and to identify gene expression changes in depressed patients. Search for biomarkers for depression using peripheral blood has attracted increasing interest in depression research, as it shares over 80% of the transcriptome with brain and is more easily accessible for research purposes (Liew et al., 2006). With this respect, proteomics and transcriptomics technologies have already been utilized in recent studies comparing the differences in protein and mRNA expression profiles in the blood of depressed patients versus healthy controls (Lee et al., 2015, Pajer et al., 2012, Xu et al., 2012). Distinct protein signatures have also been identified in post-mortem brain tissues of depressed patients (Martins-de-Souza et al., 2012).

Heterogeneity of the major depressive disorder phenotype has been recognized as one of the factors resulting in a poor replication of candidate genes for major depression (Bosker et al., 2011). General lack of understanding of the underlying neurobiology of psychiatric disorders is further hindering the identification of disease-specific biomarkers (Murck et al., 2015). Several studies have shown that the role of inflammation and HPA axis dysfunction varies depending on the subtype of depression (Anisman et al., 1999, Lamers et al., 2013, Rothermundt et al., 2001, Spanemberg et al., 2014, Yoon et al., 2012). Furthermore, depressive symptoms characterizing sickness behaviour i.e. lack of energy, sleep problems and changes in appetite, were recently shown to be independently associated with circulating levels of CRP (Jokela et al., 2015). These results suggest that the extent to which inflammation contributes to depression may be specific to the symptoms and/or to the biological correlates associated with the pathology of depression, indicating that certain type of treatment does not fit for all depressive patients. Also, many people with elevated circulating inflammatory markers and/or cortisol levels do not develop depression, suggesting that other factor(s) including stressful life events, childhood trauma, genetic predisposition, comorbid chronic diseases or lifestyle factors may play important role in the development of depressive symptoms.

**Fig. 3:**
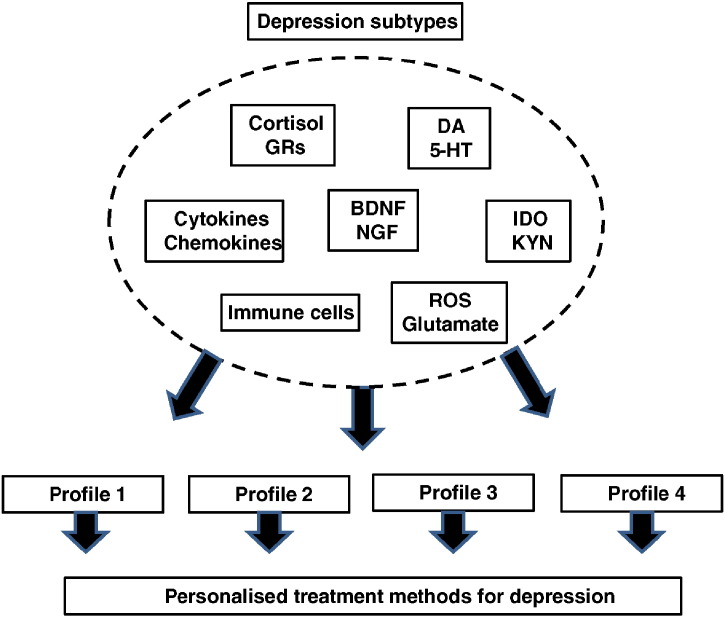
Schematic diagram showing how large-scale immunological, hormonal and neuronal characterization and personalized biological profiling of depressed subjects may advantage the development of individualized treatment methods and the identification of new biomarkers for depressive disorder.

How can omics methodologies help us to unravel the specific biological mechanisms behind the disorder in different subgroups of depressed patients and how this information may be translated into clinical practice in the future? Firstly, using conventional laboratory techniques, identification of proteins and cells being affected in different subgroups of depressed patients could be used to prescreen the potential depression-specific biomarkers (Fig. 3). Secondly, more comprehensive analyses of these markers could be exploited using omics methodologies to assess the immunological and neurohormonal profile, including expression, function and interactions between different innate and adaptive immune cells, cytokines, chemokines, costimulatory molecules, growth factors and neurohormonal mediators in the serum and/or tissues of depressed patients to identify subgroups of patients with specific biochemical signatures. Ultimately, this approach could be employed for the development depression-specific biomarkers and personalized treatment methods related to the pathological mechanisms associated with the disorder.

## 5 Conclusions and future directions

Future investigations would benefit from approaches exploring the role of the entire immune system and its relation with different neuroendocrine alterations relevant to depressive disorders. In this respect, research strategies aimed at untangling the integrative role of inflammatory cytokines, different subsets of T cells and neuroinflammatory factors in depression will be required. The combination of different technologies and the identification of different genes, proteins and metabolites related to depression neuropathology may lead to a number of breakthroughs such as the following:

1) Characterization of new biochemical pathways and neurobiological targets related to depression as well as increased understanding of their relevance for disease outcome.
2) Development of new potential biomarkers and new therapeutic applications.
3) Better understanding of individual variation in the outcome of antidepressant medication before treatment trials using predictive biomarkers.

Multi-disciplinary cooperation and utilization of a wide array of new technological platforms are vital in clarifying the role of immune alterations and exploring innovative therapeutic applications for depressive disorders.

## Conflict of interest

The authors declare that there are no conflicts of interest.
