# Supplementary material for: Innate and adaptive immunity in the development of depression: An update on current knowledge and technological advances
Source: Prog Neuropsychopharmacol Biol Psychiatry. 2016 Apr 3;66:63–72. doi: 10.1016/j.pnpbp.2015.11.012 (PMC4736094; doi:10.1016/j.pnpbp.2015.11.012)
Supplement: Supplementary file 1 — Supplementary material. [file mmc1.docx]

Innate and adaptive immunity in the development of depression: An update on current knowledge and technological advances

Rita Haapakoski, Klaus P Ebmeier, Harri Alenius, Mika Kivimäki

***Supplementary Material***

**Reference list for Figure 1:**

1. Dantzer, R. 2001. Cytokine-induced sickness behavior: where do we stand? Brain, behavior, and immunity 15:7-24.

Dantzer, R., J.C. O'Connor, G.G. Freund, R.W. Johnson, and K.W. Kelley. 2008. From inflammation to sickness and depression: when the immune system subjugates the brain. Nature reviews. Neuroscience 9:46-56.

Raison, C.L., M. Demetrashvili, L. Capuron, and A.H. Miller. 2005. Neuropsychiatric adverse effects of interferon-alpha: recognition and management. CNS drugs 19:105- 123.

2. Ulrich-Lai, Y.M., and J.P. Herman. 2009. Neural regulation of endocrine and autonomic stress responses. Nature reviews. Neuroscience 10:397-409.

Pace, T.W., and A.H. Miller. 2009. Cytokines and glucocorticoid receptor signaling. Relevance to major depression. Annals of the New York Academy of Sciences 1179:86-105.

3-4. Zorrilla, E.P., L. Luborsky, J.R. McKay, R. Rosenthal, A. Houldin, A. Tax, R. McCorkle, D.A. Seligman, and K. Schmidt. 2001. The relationship of depression and stressors to immunological assays: a meta-analytic review. Brain, behavior, and immunity 15:199-226.

5-6. Zorrilla, E.P., L. Luborsky, J.R. McKay, R. Rosenthal, A. Houldin, A. Tax, R. McCorkle, D.A. Seligman, and K. Schmidt. 2001. The relationship of depression and stressors to immunological assays: a meta-analytic review. Brain, behavior, and immunity 15:199-226.

Liu, Y., R.C. Ho, and A. Mak. 2012. Interleukin (IL)-6, tumour necrosis factor alpha (TNF-alpha) and soluble interleukin-2 receptors (sIL-2R) are elevated in patients with major depressive disorder: a meta-analysis and meta-regression. Journal of affective disorders 139:230-239.

Pavon, L., G. Sandoval-Lopez, M. Eugenia Hernandez, F. Loria, I. Estrada, M. Perez, J. Moreno, U. Avila, P. Leff, B. Anton, and G. Heinze. 2006. Th2 cytokine response in Major Depressive Disorder patients before treatment. Journal of neuroimmunology 172:156-165.

Robertson, M.J., R.S. Schacterle, G.A. Mackin, S.N. Wilson, K.L. Bloomingdale, J. Ritz, and A.L. Komaroff. 2005. Lymphocyte subset differences in patients with chronic fatigue syndrome, multiple sclerosis and major depression. Clinical and experimental immunology 141:326-332.

Pavon, L., G. Sandoval-Lopez, M. Eugenia Hernandez, F. Loria, I. Estrada, M. Perez, J. Moreno, U. Avila, P. Leff, B. Anton, and G. Heinze. 2006. Th2 cytokine response in Major Depressive Disorder patients before treatment. Journal of neuroimmunology 172:156-165.

Li, Y., B. Xiao, W. Qiu, L. Yang, B. Hu, X. Tian, and H. Yang. 2010b. Altered expression of CD4(+)CD25(+) regulatory T cells and its 5-HT(1a) receptor in patients with major depression disorder. Journal of affective disorders 124:68-75

7. Lewitus, G.M., H. Cohen, and M. Schwartz. 2008. Reducing post-traumatic anxiety by immunization. Brain, behavior, and immunity 22:1108-1114.

Lewitus, G.M., A. Wilf-Yarkoni, Y. Ziv, M. Shabat-Simon, R. Gersner, A. Zangen, and M. Schwartz. 2009. Vaccination as a novel approach for treating depressive behavior. Biological psychiatry 65:283-288.

Rook, G.A., C.A. Lowry, and C.L. Raison. 2011. Lymphocytes in neuroprotection, cognition and emotion: is intolerance really the answer? Brain, behavior, and immunity 25:591-601.

Miller, A.H. 2010. Depression and immunity: a role for T cells? Brain, behavior, and immunity 24:1-8.

8. Raison, C.L., R. Dantzer, K.W. Kelley, M.A. Lawson, B.J. Woolwine, G. Vogt, J.R. Spivey, K. Saito, and A.H. Miller. 2010. CSF concentrations of brain tryptophan and kynurenines during immune stimulation with IFN-alpha: relationship to CNS immune responses and depression. Molecular psychiatry 15:393-403.

Vecsei, L., L. Szalardy, F. Fulop, and J. Toldi. 2013. Kynurenines in the CNS: recent advances and new questions. Nature reviews. Drug discovery 12:64-82.

O'Connor, J.C., M.A. Lawson, C. Andre, M. Moreau, J. Lestage, N. Castanon, K.W. Kelley, and R. Dantzer. 2009. Lipopolysaccharide-induced depressive-like behavior is mediated by indoleamine 2,3-dioxygenase activation in mice. Molecular psychiatry 14:511-522.

9. Walsh, J.G., D.A. Muruve, and C. Power. 2014. Inflammasomes in the CNS. Nature reviews. Neuroscience 15:84-97

Alcocer-Gomez, E., M. de Miguel, N. Casas-Barquero, J. Nunez-Vasco, J.A. Sanchez- Alcazar, A. Fernandez-Rodriguez, and M.D. Cordero. 2014. NLRP3 inflammasome is activated in mononuclear blood cells from patients with major depressive disorder. Brain, behavior, and immunity 36:111-117.

Zhang, Y., L. Liu, Y.L. Peng, Y.Z. Liu, T.Y. Wu, X.L. Shen, J.R. Zhou, D.Y. Sun, A.J. Huang, X. Wang, Y.X. Wang, and C.L. Jiang. 2014. Involvement of inflammasome activation in lipopolysaccharide-induced mice depressive-like behaviors. CNS neuroscience & therapeutics 20:119-124.

10. Raison, C.L., R. Dantzer, K.W. Kelley, M.A. Lawson, B.J. Woolwine, G. Vogt, J.R. Spivey, K. Saito, and A.H. Miller. 2010. CSF concentrations of brain tryptophan and kynurenines during immune stimulation with IFN-alpha: relationship to CNS immune responses and depression. Molecular psychiatry 15:393-403.

Steiner, J., M. Walter, T. Gos, G.J. Guillemin, H.G. Bernstein, Z. Sarnyai, C. Mawrin, R. Brisch, H. Bielau, L. Meyer zu Schwabedissen, B. Bogerts, and A.M. Myint. 2011. Severe depression is associated with increased microglial quinolinic acid in subregions of the anterior cingulate gyrus: evidence for an immune-modulated glutamatergic neurotransmission? Journal of neuroinflammation 8:94.

Hashimoto, K., A. Sawa, and M. Iyo. 2007. Increased levels of glutamate in brains from patients with mood disorders. Biological psychiatry 62:1310-1316.

Shelton, R.C., J. Claiborne, M. Sidoryk-Wegrzynowicz, R. Reddy, M. Aschner, D.A. Lewis, and K. Mirnics. 2011. Altered expression of genes involved in inflammation and apoptosis in frontal cortex in major depression. Molecular psychiatry 16:751- 762.

Savitz, J., W.C. Drevets, C.M. Smith, T.A. Victor, B.E. Wurfel, P.S. Bellgowan, J. Bodurka, T.K. Teague, and R. Dantzer. 2015. Putative neuroprotective and neurotoxic kynurenine pathway metabolites are associated with hippocampal and amygdalar volumes in subjects with major depressive disorder. Neuropsychopharmacology : official publication of the American College of Neuropsychopharmacology 40:463- 471.

Bocchio-Chiavetto, L., V. Bagnardi, R. Zanardini, R. Molteni, M.G. Nielsen, A. Placentino, C. Giovannini, L. Rillosi, M. Ventriglia, M.A. Riva, and M. Gennarelli. 2010. Serum and plasma BDNF levels in major depression: a replication study and meta- analyses. The world journal of biological psychiatry : the official journal of the World Federation of Societies of Biological Psychiatry 11:763-773.

Bondy, B., T.C. Baghai, C. Minov, C. Schule, M.J. Schwarz, P. Zwanzger, R. Rupprecht, and H.J. Moller. 2003. Substance P serum levels are increased in major depression: preliminary results. Biological psychiatry 53:538-542.

Dantzer, R., J.C. O'Connor, M.A. Lawson, and K.W. Kelley. 2011. Inflammation- associated depression: from serotonin to kynurenine. Psychoneuroendocrinology 36:426-436.

11. Xanthos, D.N., and J. Sandkuhler. 2014. Neurogenic neuroinflammation: inflammatory CNS reactions in response to neuronal activity. Nature reviews. Neuroscience 15:43-53.

Tilleux, S., and E. Hermans. 2007. Neuroinflammation and regulation of glial glutamate uptake in neurological disorders. Journal of neuroscience research 85:2059-2070.

Setiawan, E., A.A. Wilson, R. Mizrahi, P.M. Rusjan, L. Miler, G. Rajkowska, I. Suridjan, J.L. Kennedy, P.V. Rekkas, S. Houle, and J.H. Meyer. 2015. Role of translocator protein density, a marker of neuroinflammation, in the brain during major depressive episodes. JAMA psychiatry 72:268-275.

**Abbreviations for Figure 1**:

ACTH: adrenocorticotropic hormone; ANS: autonomic nervous system; BDNF: brain-derived neurotropic factor; CRH: corticotrophin releasing hormone; DA: dopamine; DC: dendritic cell; E: epinephrine; 5-HT: 5-hydroxytryptamine; IDO: indoleamine-2,3-dioxygenase; HPA axis: hypothalamic–pituitary–adrenal axis; Ig: immunoglobulin; IL: interleukin; NE: norepinephrine; NK cell: natural killer cell; PRR: pattern recognition receptor; ROS: reactive oxygen species; SP: substance P; TRYCAT: tryptophan catabolite; KYN: kynurenine.
